# Folic acid supplementation on inflammation and homocysteine in type 2 diabetes mellitus: systematic review and meta-analysis of randomized controlled trials

**DOI:** 10.1038/s41387-024-00282-6

**Published:** 2024-04-22

**Authors:** Kabelo Mokgalaboni, Given. R. Mashaba, Wendy N. Phoswa, Sogolo. L. Lebelo

**Affiliations:** Department of Life and Consumer Science, College of Agriculture and Environmental Sciences, University of South Africa, Florida Campus, Roodepoort, South Africa

**Keywords:** Cardiovascular diseases, Type 2 diabetes

## Abstract

**Background:**

The beneficial effects of folate have been observed under different conditions, but the available evidence on inflammation and reduction of cardiovascular disease (CVD) in type 2 diabetes mellitus (T2DM) is limited. The study aimed to explore the effects of folate on inflammation and homocysteine amongst individuals with T2DM.

**Methods:**

PubMed, Scopus, and Cochrane Library were used to search for evidence. A random-effect model meta-analysis through Review Manager (version 5.4) and metaHun was performed. Results were reported as standardized mean differences (SMD) and 95% confidence intervals graphically using forest and funnel plots.

**Results:**

Data from 9 trials with 426 patients living with T2DM were analyzed. Folic acid supplementation significantly revealed a large effect size on homocysteine levels compared to placebo, SMD = −1.53, 95%CI (−2.14,−0.93), *p* < 0.05. Additionally, we observed a medium marginal effect size on C-reactive protein (SMD = −0.68, 95%CI (−1.34, −0.01), *p* = 0.05). However, no significant effect on tumor necrosis factor-α (SMD = −0.86, 95%CI (−2.65, 0.93), *p* = 0.34), and interleukin-6 (SMD = −0.04, 95%CI (−1.08, 1.01), *p* = 0.95) was observed.

**Conclusion:**

Evidence analyzed in this study suggests that folic acid supplementation in T2DM reduces homocysteine and may mitigate CVDs. However, its effect on inflammation is inconclusive.

## Introduction

Type 2 diabetes mellitus (T2DM) is a condition that elevates blood glucose, also known as hyperglycemia [1]. Patients living with T2DM are at higher risk of developing cardiovascular diseases (CVD) than healthy individuals [2]. The prevalence of diabetes worldwide in 2021 was estimated to be 10.3% and this is continually rising with estimated projections of around 12.2% in 2045 [3]. The main factors that exacerbate the development of CVD in T2DM include but are not limited to hyperinsulinemia, obesity, hypertension, hypertriglyceridemia, hypercholesterolemia, and homocysteinemia [4]. Inflammation is a central feature in T2DM, contributing to CVD among patients with T2DM. For instance, an increased circulating plasma C-reactive protein (CRP) [5, 6] is noted in patients with T2DM, which may further increase the risk of CVD. Moreover, T2DM are more likely to develop hyperhomocysteinemia primarily due to folate and vitamin B_12_ deficiency [7, 8]. It is noteworthy to indicate that patients with T2DM who rely on metformin to control hyperglycemia often develop hyperhomocysteinemia, which further makes them susceptible to CVDs [9].

Previous studies have reported a link between homocysteine and inflammation [10–12]. For instance, evidence from preclinical and clinical studies demonstrated an association between homocysteine and proinflammatory responses [10, 13]. Homocysteine is an amino acid associated with the risk of CVD if its level is elevated in the body [14]. Although homocysteine and inflammation markers are frequently detected at the same time, they are not correlated as they have revealed an inverse relationship in previous scientific evidence [8, 15]. We anticipate that measuring them simultaneously will improve the overall interpretation and understanding of their correlation in T2DM. Due to their contribution to the development of CVDs in T2DM, an approach that can reduce the circulating levels of inflammatory markers and homocysteine in T2DM can be important to ameliorate inflammation, halt cardiovascular-related complications amongst T2DM and further reduce morbidity and mortality. It is important to note that T2DM medications widely used to control hyperglycemia in T2DM are available; however, their long-term may result in vitamin B_12_ and folate deficiency [16, 17]. Recent evidence has shown that folate deficiency is associated with an increased level of homocysteine, increasing the risk of CVD in T2DM [18, 19]. The above shortfalls for drugs and related side effects have prompted an exploration of dietary supplements and macronutrients in alleviating cardiovascular-related complications in T2DM. Other examples include vitamin D [20], and folate, a natural form of vitamin B_9_ due to its pleiotropic effects in diabetes and fewer side effects than other expensive and toxic therapies [21]. Folate is primarily found in green leafy vegetables and plays a role in cell division and synthesis of nucleic acids [22].

Folate has been explored by previous studies on inflammation, focusing on CRP levels in patients with T2DM. However, the findings reported by various studies are contradictory [23, 24]. While Fatahi et al. [25] through meta-analysis, demonstrated a positive effect of folate on inflammation, the results must be treated with caution as only CRP as a marker of inflammation was assessed, and this might introduce bias, as it is difficult to conclude on the severity of inflammation based on one biomarker. Sato has reported no effect of 20 mg of folic acid on CRP and IL-6 as markers of inflammation in T2DM [15]. Additionally, the findings by Kaye et al., [26] suggest that folic acid may reduce the homocysteine levels in T2DM. However, completely different results were reported in a randomized, placebo-controlled, cross-over trial where there was no significant difference between folic acid and placebo groups on homocysteine [27]. Thus, the current study aims to systematically review and meta-analyze data from RCT to evaluate the effect of folic acid/folate on the level of homocysteine and inflammation in T2DM adult patients to rule out any inconsistencies observed in previous evidence. Furthermore, this review and meta-analysis also indicate the effective doses of folic acid/folate therapy that can alleviate inflammation among patients living with T2DM.

## Methodology

This systematic review and meta-analysis are prepared and reported using an updated Preferred Reporting Items for Systematic Review and Meta-Analysis guideline [28] and checklist (Appendix 1). The protocol for this study has been registered with PROSPERO (the International Prospective Register of Systematic Reviews), CRD2023476986, for transparency.

### Aim of the study

To evaluate if folic acid supplementation can ameliorate inflammation. In addition, we sought to determine the overall effect of folic acid on homocysteine in T2DM.

### Information source and search strategy

A comprehensive literature search was conducted using online databases, including PubMed, Scopus, and Cochrane Library. The following medical subject headings (MeSH) and keywords were used: “Folate” OR “folic acid” OR “Folacin” OR “Vitamin B_9_” AND “type 2 diabetes mellitus” OR “type 2 diabetes” OR “hyperglycemia.” These search terms were adjusted to suit each database used. The search was restricted to randomized controlled trials published from inception until 15 October 2023. The search was also restricted to RCT published in English. Furthermore, the reference lists of retrieved studies were also screened for additional relevant studies. Two researchers (KM and GRM) independently screened titles and abstracts of all retrieved studies, and discrepancies were resolved through discussion and re-evaluation of the study.

### Eligibility criteria

#### Inclusion criteria

All trials that satisfied our PICOS criteria were included (Table 1); for instance, all participants were adult patients living with T2DM, folic acid or folate treatment as an intervention, placebo as a control group, and inflammation and homocysteine levels as the outcomes of interest. Therefore, all randomized controlled trials that evaluated the effect of folic acid on concentrations of homocysteine, CRP, IL-6, and TNF-α were included.

**Table 1:** Eligibility criteria according to PICOS.

PICOS	Population/Participants of interest	Adults (18+ years) with type 2 diabetes mellitus
	Intervention	Folate/folic acid/vitamin B_9_ for a period of 2 to 26 weeks
	Comparator/control	Placebo/standard treatment
	Outcomes	Changes in homocysteine and inflammation
	Study design	All Randomized controlled trials (cross-over, parallel, open-labeled, single and double-blinded)

*PICOS* population, intervention, comparator/control, outcome and study design.

### Exclusion criteria

In case of any unavailable full-text article, we contacted the corresponding author, and if there was no response, the paper was excluded. Clinical trials using other interventions were excluded: no information about inflammation, no control groups, pregnant women, animal models of T2DM, gray literature, or other supplements were excluded.

### Data items and extraction

The main researcher (KM) designed an Excel extraction sheet, and this was shared with the secondary researcher GRM prior to extraction. This Excel sheet included data items such as leading author surname, year of publication, study location, study design, population, gender distribution, mean age, mean body mass index, glycated hemoglobin, method of determining homocysteine, study duration, dosage of folic acid supplements, as the means ± standard deviation (SD) of the outcome measures in the folate/folic acid and placebo groups at baseline and post-intervention (change in values) for inflammatory markers (CRP, IL-6, and TNF-α) and homocysteine levels. The data were extracted independently by KM and GRM to minimize the risk of bias and extraction errors. Any disagreement was resolved by a third independent researcher WNP, who re-evaluated the study or data items in question.

### Risk of bias in individual studies

The risk of bias (ROB) across all eligible studies was assessed following the Cochrane risk of bias tool [29]. Each included trial was evaluated based on five domains: bias arising from the randomization process, bias due to deviation from intended intervention, bias due to missing outcome data, bias due to missing outcome data, bias in the measurement of the outcome, bias in the selection of the reported results. A study was judged as low risk if all domains were at low risk of bias and judged as some concern if one domain was judged as some concern. Two independent researchers (KM and GRM) made the overall judgment. In the event of disagreement, a third researcher (WNP) made a judgment of the domain in question.

### Data synthesis and statistical analysis

The meta-analysis was carried out using Review Manager (RevMan Version 5.4) and Meta-Hun http://softmed.hacettepe.edu.tr/metaHUN/ (accessed on 28/10/23). Means and SD of the outcome measures (CRP, IL-6, TNF-α, and homocysteine) reported for the folate and placebo groups were used to obtain the overall estimates. Where the SD of the mean difference was not reported in the studies, we estimated it using the following formula: change in SD = √[(SD pre-treatment)^2^ + (SD post-treatment)^2^ – (2 R × SD pre-treatment × SD post-treatment)]. The correlation coefficient (R) of 0.8 was considered based on previous reports [30]. Additionally, the mean was estimated using formulae ${\rm{X}}{\rm{\bar{} }}=\frac{{\rm{a}}+2{\rm{m}}+{\rm{b}}}{4}$, and SD was estimated using ${\rm{SD}}=\frac{{\rm{range}}}{4}$ when the sample size is smaller than 70 if the trial reported median and interquartile range (IQR) [31]. In contrast, SD was estimated using the standard error of the mean (SEM) $={\rm{SD}}\div\surd {\rm{n}}$ when the trial reported a standard error of the mean. The overall effect sizes for all effect measures were reported as standardized mean differences (SMD) and 95% confidence intervals (CI). SMD was preferred due to variables being reported in different units of measure. Random-effect model was used due to moderate heterogeneity, respectively. We further assessed heterogeneity between studies through the *I*^*2*^ statics test (*I*^*2*^ > 50%), considered as moderate heterogeneity [32, 33]. A *p*-value of < 0.05 was considered statistically significant, and the Cohens d was used to interpret the magnitude of the effect size. A Cohens d of 0.1, 05, and 0.8 was classified as a small, medium, and large effect, respectively. Publication bias was evaluated graphically through funnel plot inspection and statistically using Egger’s regression test. A sensitivity analysis was conducted using a leave-one-out analysis to determine each study’s effect on the overall effect size [34, 35]. The overall certainty of evidence across the studies was evaluated in accordance with the Grading of Recommendations Assessment, Development, and Evaluation (GRADE) guideline [36]. The quality of evidence was classified into four categories based on the corresponding evaluation criteria: high, moderate, low, and very low.

## Results

### Characteristics of included studies

Nine trials [23, 24, 27, 37–42] with 426 T2DM on folate/folic acid compared to placebo were included. The intervention group had 226 patients in the T2DM compared to 201 in the placebo group. These trials were conducted in Australia [40], Canada [27], India [38], Iran [24, 38, 39, 41], Egypt [37], Norway [42] and the Netherlands [23] between 1998 and 2022. All included trials were randomized controlled trials, with one open-labeled [38], one double-blinded cross-over [27], and the rest were double-blinded trials. The sample size varied from smallest (26) to largest (100). The treatment was administered as either folate/folic acid in all trials between doses of 0.25 mg to 10 mg for a period ranging from 2 weeks to 28 weeks. At least seven trials [23, 24, 37–41] used folate at 5 mg for 4–26 weeks. The mean age of participants in the folate group was 59.57 ± 4.68 years, with a body mass index of 27.39 ± 3.05 kg/m^2^. The baseline glycated hemoglobin in the folate group was 7.67 ± 0.40%. The gender distribution across all trials was 134 males in folate and 130 in the placebo group, respectively. Different techniques were used to measure homocysteine in these trials, with the common one being an Enzyme immunoassay by homocysteine kit followed by an enzyme-linked immunosorbent assay (ELISA) kit. A detailed overview of the included trials is presented in Table 2.

**Table 2:** General overview characteristics of included nine trials.

Study and design	Country	Population	Intervention	Age (Years)	Gender (male %)	BMI (kg/m^2^)	HBA1C (%)	Reported effect measures	Method of homocysteine determination
Aarsand and Carlsen, [42] Prospective randomized, double-blinded controlled trial	Norway	28 type 2 diabetes mellitus (T2DM), 14 on folate and 14 on placebo	Folate at 0.25 mg for 12 weeks	61.6 ± 9.35 56.7 ± 10.47	10 (71.4) 11 (78.6)	29.2 ± 5.24 28.3 ± 4.11	NR	Homocysteine	Automated high-performance liquid chromatography (HPLC)
Spoelstra-de Man et al. [23] Randomized, double-blind, controlled trial	Netherlands	41 T2DM, 23 on folic acid and 18 on placebo	Folic acid at 5 mg for six months	63.7 ± 8.6 66.1 ± 8.5	14 (61) 10 (56)	29.3 ± 3.9 28.8 ± 3.4	7.6 ± 1.3 7.3 ± 1.2	Homocysteine C-reactive protein (CRP) Interleukin-6 (IL-6) Tumor necrosis factor-alpha (TNF-α)	HPLC
Mangoni et al. [40] Parallel, randomized, double-blinded controlled trial	Australia	Twenty-six patients with T2DM, 13 on folic acid 13 on placebo	Folic acid at 5 mg for 4 weeks	55.3 ± 1.2 57.6 ± 1.3	8 (61.5) 6 (46.2)	30.5 ± 1.1 32.3 ± 1.8	8.3 ± 0.5 8.3 ± 0.4	Homocysteine	Fluorescence polarization immunoassay on an IMX analyzer
Aghamohammadi et al. [41] Randomized, double-blinded controlled clinical trial	Iran	68 T2DM, 34 on folic acid 34 placebo	Folic acid at 5 mg for 8 weeks	58.72 ± 6 7.2 55.6 ± 6 9.3	34 (100) 34 (100)	27.4 ± 3.2 27.8 ± 6 4	7.5 ± 1.5 7.6 ± 1.4	Homocysteine	Enzyme immunoassay by homocysteine kit
Gargari et al. [39] Randomized, double-blinded controlled clinical trial	Iran	48 T2DM, 24 on folic acid 24 placebo	Folic acid at 5 mg for eight weeks.	59.4 ± 7.6 57 ± 10.1	24 (100) 24 (100)	28.8 ± 2.7 28.5 ± 3.3	7.5 ± 1.5 7.7 ± 1.6	Homocysteine	Enzyme immunoassay method by homocysteine kit
Title et al. [27] Randomized, double-blinded placebo-controlled, cross-over trial	Canada	38 T2DM, 19 folic acid 19 placebo	Folic acid at 10 mg for 2 weeks	54.5 ± 5.9	9 (47.4)	NR	NR	Homocysteine hs-CRP TNF-α	Automated analyzers
Talari et al. [24] Randomized, double-blind, placebo-controlled trial	Iran	60 T2DM, 30 on folate 30 on placebo	Folate at 5 mg for 12 weeks	62.1 ± 9.6 65.4 ± 11.5	13 (43.3) 13 (43.3)	29.8 ± 3.8 29.8 ± 4.4	NR	Homocysteine hs-CRP	Enzyme immunoassay by homocysteine kit
Satapathy et al. [38] Randomized, multi-arm, open-label clinical trial	India	37 T2DM, 18 on folic acid 19 on placebo	Folic acid at 5 mg for 8 weeks	53.64 ± 5.63 54.28 ± 6.41	NR	25.82 ± 3.22 24.85 ± 2.88	7.54 ± 1.13 7.75 ± 1.36	Homocysteine TNF-α IL-6 hs-CRP	Enzyme-linked immunosorbent Assay (ELISA) kits
El-khodary et al. [37] Randomized, double-blinded controlled clinical trial	Egypt	100 T2DM, 50 on folic acid 50 on placebo	Folic acid at 5 mg for 12 weeks	52.86 ± 9.46 54.46 ± 7.81	22 (44) 23 (46)	28.34 ± 3.11 27.69 ± 2.64	7.57 ± 0.79 7.56 ± 0.76	Homocysteine hs-CRP	ELISA kits

*T2DM* type 2 diabetes mellitus, *hs-CRP* high sensitivity C-reactive protein, *NR* not reported, *BMI* body mass index, *HBA1C* glycated hemoglobin, *HPLC* high-performance liquid chromatography, *IL-6* interleukin-6, *TNF-α* tumor necrosis factor-alpha.

### Literature search

**Fig. 1:**
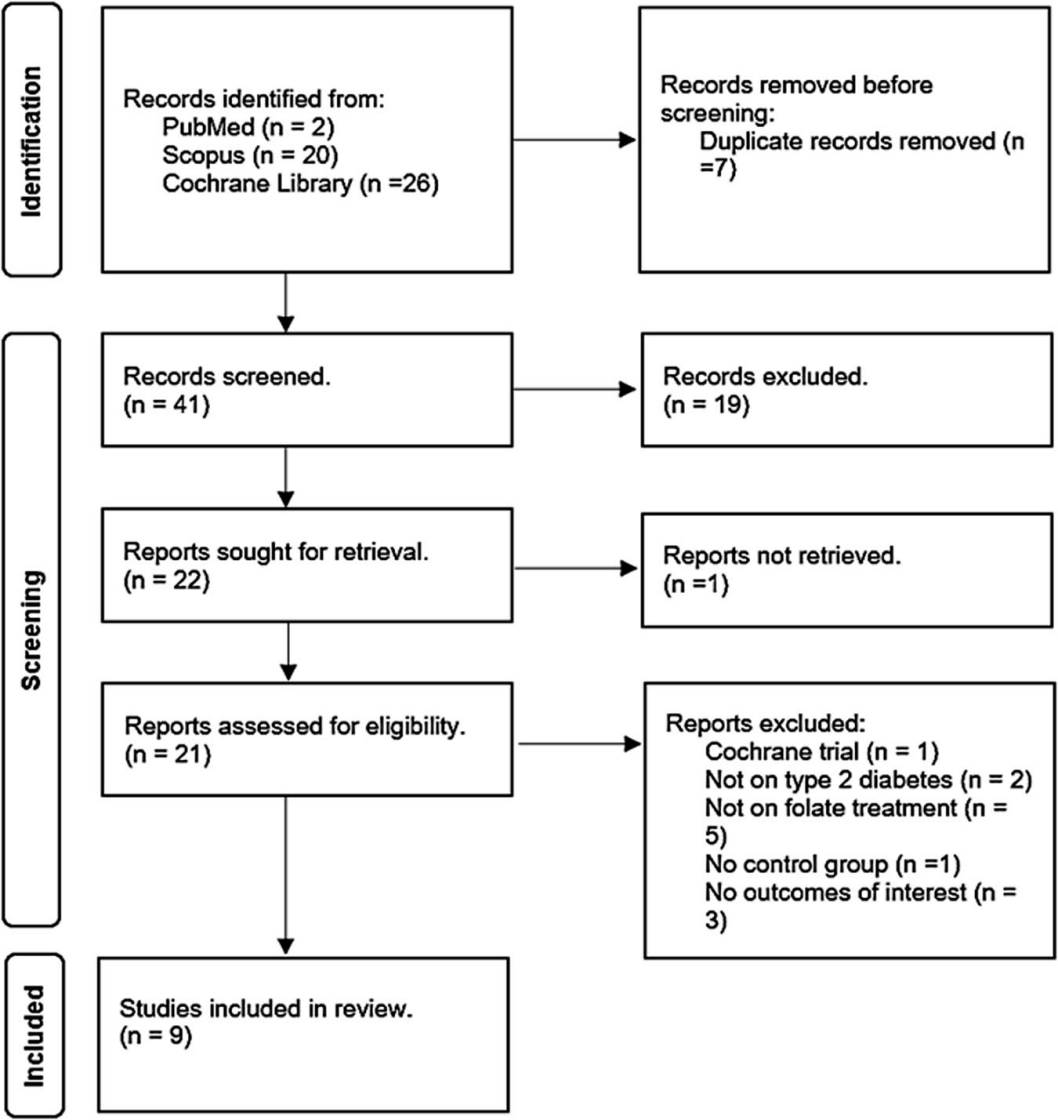
Flow diagram showing study selection.

A comprehensive overview of the study selection process is presented in the PRISMA flow diagram in Fig. 1. The electronic search across three main databases, namely PubMed, Scopus, and the Cochrane Library, identified forty-eight records. PubMed contributed two records, Scopus contributed twenty, and the Cochrane Library contributed twenty-six (Table 1S). All forty-eight records were stored in the Mendeley Reference Manager (version 2.104.0). Duplicate identification revealed seven records replicated in the databases and were excluded. Consequently, forty-one unique records underwent screening by independent researchers (KM and GRM). During the initial screening based on eligibility criteria, nineteen records were deemed irrelevant due to unrelated titles, abstracts, keywords, and focus.

Among the remaining twenty-two records subjected to full screening, one was published in Chinese and was excluded to prevent potential misinterpretation upon translation to English. Of the remaining twenty-one records, five employed interventions other than folate; three did not address our specified outcomes of interest, two involved irrelevant populations as they were not T2DM, one did not have a control group, and one represented a registration for an ongoing Cochrane trial. Consequently, only nine trials met the eligibility criteria for relevance to our study.

### Effect of folic acid supplementation on homocysteine

**Fig. 2:**
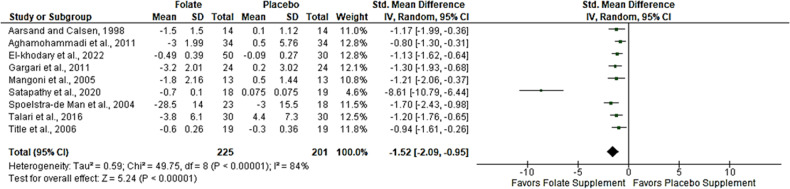
Effect of folic acid supplementation on homocysteine in T2DM.

The effect of folic acid supplementation on homocysteine was analyzed from nine trials [23, 24, 27, 37–42] with a sample size of 426 T2DM on folic acid versus placebo. The overall effect from the random effect model showed a large effect size demonstrated by a significant reduction in the level of homocysteine in T2DM on folic acid compared to placebo, SMD = −1.53, 95%CI (−2.14,−0.93), *p* < 0.05 (Fig. 2). Of concern was an observed level of heterogeneity (*I*^*2*^ = 86%) across the included studies.

### Effect of folic acid supplementation on inflammation

**Fig. 3:**
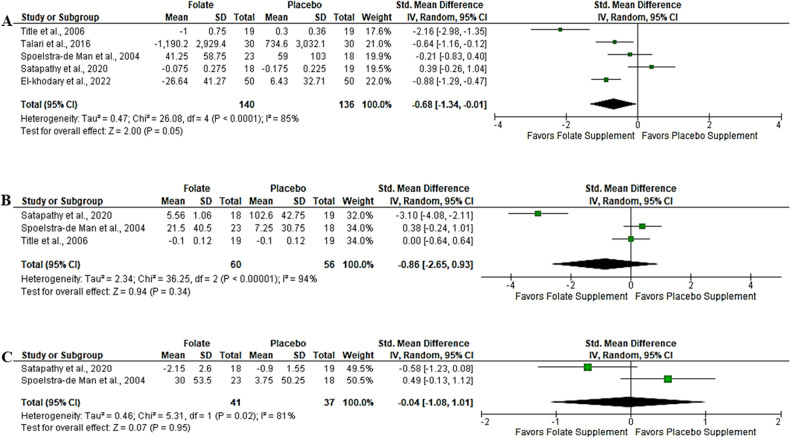
Effect of folic acid supplementation on inflammation. **A** high sensitive-C-reactive protein (hs-CRP); **B** Tumor necrosis factor alpha (TNF-α); **C** Interleukin-6 (IL-6).

Three markers of inflammation were evaluated: CRP, TNF-α, and IL-6; however, only five trials [21, 25, 35–37] evaluated CRP in T2DM following folic acid supplementation compared to placebo. The results from the random-effect meta-analysis revealed a medium effect size demonstrated by a marginal reduction in CRP; SMD = −0.68, 95%CI (−1.34, −0.01), *p* = 0.05 (Fig. 3A). This was accompanied by a high level of heterogeneity (*I*^*2*^ = 85%). Likewise, the results from 3 trials [23, 27, 38] that evaluated TNF-α showed a large effect size; however, this was statistically not significant, SMD = −0.86, 95%CI (−2.65, 0.93), *p* = 0.34 (Fig. 3B). These trials revealed moderate evidence of heterogeneity (*I*^*2*^ = 94%). Only two trials [23, 38] assessed the effect of folate on IL-6; the evidence from the random-effect meta-analysis showed a small effect size, and this also not significant, SMD = −0.04, 95%CI (−1.08, 1.01), *p* = 0.95 (Fig. 3C). Similarly, there was moderate evidence of heterogeneity across these trials (*I*^*2*^ = 81%).

### Heterogeneity and subgroup analysis

Due to observed heterogeneity in homocysteine results, we performed subgroup analyses based on both sample size (< or > 50 T2DM patients) and folate dosage (0.25, 5, or 10 mg). The subgroup analysis of the sample size revealed evidence of heterogeneity (*I*^*2*^ = 70.5%) (Fig. 1SA). Notably, studies with a sample size below 50 exhibited high heterogeneity (*I*^*2*^ = 89%), contrasting with those above 50, which displayed no heterogeneity (*I*^*2*^ = 0%) (Fig. 1SA).

Additionally, the subgroup analysis on the dosage of folic acid supplementation showed a decrease in heterogeneity (*I*^*2*^ = 20.1%); however, a 5 mg administration led to a 2% decrease in heterogeneity (*I*^*2*^ = 86%) (Fig. 1SB). Subgroup analysis on duration of intervention revealed reduced heterogeneity (I^2^ = 35.8%). Interestingly, medium duration demonstrated no evidence of heterogeneity (*I*^*2*^ = 0%) (Fig. 2SA). Finally, a subgroup analysis based on gender distribution for homocysteine revealed an overall test difference indicating heterogeneity (*I*^*2*^ = 95.7%). However, studies conducted in males showed no heterogeneity(*I*^*2*^ = 0%), whereas those involving both genders resulted in high heterogeneity (*I*^*2*^ = 89%) (Fig. 2SB). The results of a meta-analysis on CRP revealed a moderate heterogeneity(*I*^*2*^ = 73%). This warranted subgroup to find the source of this variation; firstly, we subgrouped studies according to sample size and dosage of folic acid supplementation. We found that the evidence from trials with a sample size below 50 slightly changed heterogeneity (*I*^*2*^ = 50%), while more than 50 had *I*^*2*^ = 0% (Fig. 3SA). Interestingly, the overall test for subgroup differences also revealed no heterogeneity (*I*^*2*^ = 0). On the other hand, subgroups according to dosage showed that trials that used 5 mg of folate/folic acid had *I*^*2*^ = 74%, while those that used 10 mg had *I*^*2*^ = 85%. Moreover, the test for subgroup difference showed *I*^*2*^ = 92.3% (Fig. 3SB).

### Publication bias (Funnel plots and Egger’s regression test)

**Fig. 4:**
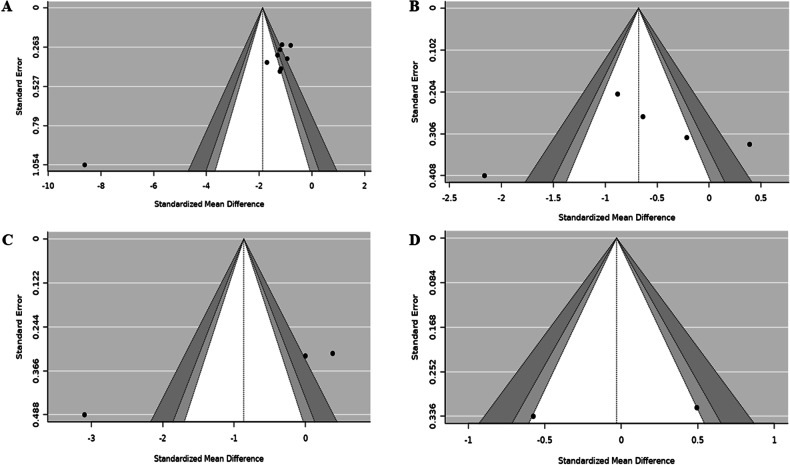
Publication bias evaluation on homocysteine and inflammatory markers. **A** Homocysteine; **B** C-reactive protein (CRP); **C** Tumor necrosis factor (TNF)-α; **D** Interleukin (IL)-6.

Publication bias was assessed by visualization of the funnel plot, and we noted evidence of bias on homocysteine (Fig. 4A), and this was consistent with the findings of the Eggers regression test (Z score = −6.06, p < 0.05). This suggests that trials with positive significant results were more likely to be published than negative trials. On the contrary, the funnel plot revealed no evidence of bias on hs-CRP- (Fig. 4B), which was supported by the Eggers regression test (Z score = −0.75, p = 0.46). For TNF-α, Eggers regression showed some potential level of bias (Z score = −6.15, p < 0.05); this was supported by a funnel plot (Fig. 4C). Additionally, IL-6 revealed no bias graphically (Fig. 4D); however, due to few trials, no regression was assessed.

### Sensitivity analysis across the trials

Sensitivity results for homocysteine showed that the exclusion of the study by Satapathy et al. [38], due to small weight, resulted in the change in overall effect size, SMD = −1.14, 95%CI (−1.36, −0.92), *p* = 0.0000 (Table 2S). The exclusion of El-khodary et al. [37] in CRP results changed the overall effect size to SMD = −0.63, 95%CI (−1.56, 0.30), p = 0.16 (Table 3S). When Satapathy et al., [38] was excluded from TNF-α analysis, the effect size changed to SMD = 0.20, 95%CI (−0.27, 0.66), *p* = 0.0000 (Table 4S). For IL-6, exclusion of [38] led to SMD = 0.49, 95%CI (−0.13, 1.12), *p* = 0.0000 (Table 5S).

### Assessment of risk of bias across and certainty of evidence across the included trials

**Fig. 5:**
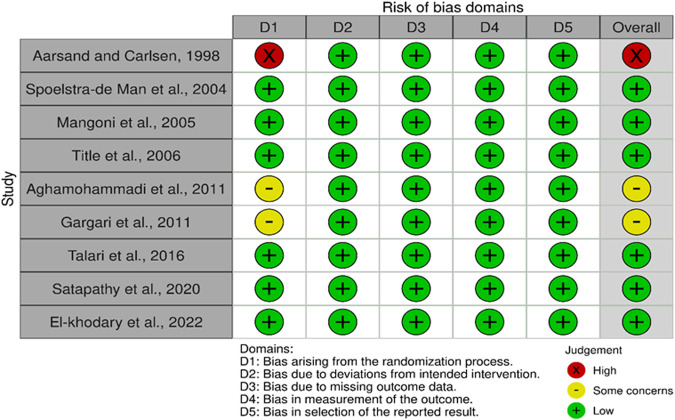
Risk of bias according to ROB tool 2.0.

Among nine trials, two trials [39, 41] were judged as having some concerns of bias as the process of randomization was not clear in their methodology. Only one trial was judged as high risk [42], due to lack of information about the randomization method used. Interestingly, 67% of trials [23, 24, 27, 37, 38, 40] were judged as low risk of bias as they scored low risk across all domains (Fig. 5). GRADING of trials revealed moderate certainty of evidence on homocysteine, CRP, and TNF-α, while IL-6 evidence was found to be of low certainty (Table 6S). The downgrade was due to heterogeneity, risk of bias, or imprecision due to the small sample size (<400).

## Discussion

To the best of our knowledge, this is the first comprehensive meta-analysis of RCT to evaluate the effect of folic acid supplementation on homocysteine and inflammation in adult patients with T2DM. We found that folic acid supplementation was associated with a reduction in homocysteine levels. The observed SMD (1.52) was large effect, suggesting anti-homocysteine properties. Additionally, there was a marginal effect of folic acid on CRP without a significant effect on TNF-α and IL-6 in patients with T2DM. Subgroup analysis showed that the folate effect on homocysteine was more pronounced at a higher dose (10 mg) than 5 mg supplementation. However, it is important to note that only one trial used 10 mg compared to 5 mg that used 5 mg of folic acid. It was evident that studies with a sufficient sample size (50 and above patients) had a more pronounced effect than those with a smaller sample size. Although folic acid supplementation at both short and medium periods reduced homocysteine, the reduction was more pronounced at short periods (0–4 weeks) compared to medium periods (8–12 weeks). Our research reveals lower homocysteine levels in individuals living with T2DM receiving folate supplements, indicating a potential decrease in the risk of CVD. We are confident with the evidence synthesized in this study as the evidence showed moderate certainty in homocysteine. In T2DM, insulin resistance and associated impaired kidney function results in an elevated homocysteine level [43–47]. This elevation promotes the development of CVD complications associated with T2DM.

Interestingly, the evidence from this study shows that folic acid supplementation can reduce homocysteine by converting it into methionine, lowering the risk of cardiovascular complications in T2DM patients [26]. Notably, previous evidence has shown that folic acid supplementation can reduce homocysteine levels in patients living with T2DM by increasing the 5-methyltetrahydrofolate intracellular pool [37]. This effect is crucial as an elevated level of homocysteine can damage blood vessels and contribute to the development of CVD [45]. Therefore, any strategies therapeutically that reduce homocysteine may assist in alleviating CVD among T2DM. In obese children, similar trends have been observed when administering a minimum of 1 g of folic acid, leading to a substantial reduction in homocysteine levels [48]. Similar findings are observed in gestational diabetes, as 1 mg and 5 mg of folic acid supplementation significantly reduced homocysteine levels [49]. Although the pathological and physiological mechanisms of these conditions differ, these findings demonstrate the efficacy of folate supplementation across diverse conditions and age groups. A non-randomized trial in menopausal T2DM women also showed that 800 µg of folate significantly reduced homocysteine levels. However, this study revealed an inverse correlation between folic acid and homocysteine (r = −0.4876, *p*-value = 0.0134) [50]. Although the mechanism by which folate reduces homocysteine in T2DM is poorly documented, it is assumed that this is associated with the role of folate in one-carbon metabolism. Folic acid supplementation increases the availability of one-carbon units, which then promotes the remethylation of homocysteine to methionine [51]. This subsequently results in a decrease in homocysteine levels in the body. While such benefits are acknowledged, contrasting findings from other studies suggest a possible risk of CVD in T2DM, even with folic acid supplementation [52]. These findings suggest a limitation in the beneficial effect of folate, especially in T2DM. It is assumed that folate deficiency impairs the conversion of homocysteine to methionine, resulting in homocysteine accumulation in the blood [53]. High homocysteine levels are associated with an increased risk of CVDs and other health problems.

Although there was a marginal effect on hs-CRP (*p* = 0.05), no significant effect of folic acid supplementation on other markers of inflammation was observed. This was shown by no significant effect on TNF-α and IL-6 following supplementation with folic acid compared to placebo. A reduction in hs-CRP following folic acid supplementation reveals, to some extent, the beneficial effect of folic acid as an anti-inflammatory agent. However, as not all inflammatory markers were reduced, the findings are thus inconclusive. Among some factors contributing to the challenge in elucidating conflicting findings regarding the impact of folic acid on inflammation is the limited number of trials conducted. The inability of folic acid to improve some markers of inflammation indicates that it does not exhibit anti-inflammatory properties. For instance, Spoelstra-de Man et al., [23] reported no effect of folic acid on hs-CRP, IL-6, and TNF-α. The same findings were observed by Title et al. [27], however, only TNF-α and hs-CRP were investigated, and no effect was observed. Despite these null findings, another trial observed a significant effect of folic acid on hs-CRP in T2DM, as demonstrated by a significant decrease in hs-CRP within the folic acid group before and after folic acid supplementation. This same trend was also observed when folic acid supplementation was compared to placebo [24]. The latter supports our findings as we observed a reduction in hs-CRP with a medium to large effect size (Cohen d = 0.68). Other researchers reported a significant decrease in IL-6 and TNF-α following folic acid supplementation compared to placebo, suggesting the anti-inflammatory effects of folic acid in T2DM [38]. These findings differ from our overall findings in this study as we reported no significant effect of folic acid on TNF-α and IL-6. Another study showed a significant change between baseline and post-treatment on hs-CRP, however, there were no significant changes between the folic acid and placebo groups [38]. In obese children, 1 mg of folic acid has proven to offer an anti-inflammatory effect, as demonstrated by a significant decrease in IL-6, TNF-α, and IL-8 [48].

Similarly, El-khodary et al. [37] also showed a significant decrease in hs-CRP between baseline and post-treatment (*p* = 0.008). The same trial reported a significant decrease in hs-CRP following three months of folic acid supplementation compared to placebo (*p* = 0.005). This study also showed a positive correlation between homocysteine and hs-CRP (r = 0.308, *p* = 0.002). Due to these contradicting results on inflammation, the effect of folic acid on inflammation is not clear, other researchers have suggested that folic acid may be involved in the reduction of hs-CRP by reducing homocysteine and oxidative stress. For instance, Talari et al., [24] reported an increased adjusted glutathione (GSH) following folic acid supplementation in T2DM compared to placebo.

Additionally, folic acid exhibits anti-insulinemic activities [54, 55] may further alleviate inflammation by suppressing the synthesis of inflammatory cytokines. It is important to note that while the benefits were not observed in this study, this might be attributable to the number of trials analyzed primarily because observational differences were noted. Previous evidence suggests that homocysteine promotes the expression of inflammatory markers by increasing the activation of nuclear factor kappa β (NF-kβ) and poly-adenosine diphosphate (ADP) ribose polymerase. Therefore, we speculate that folic acid anti-homocysteine properties may alleviate inflammation by inhibiting the activation of NF-κβ and ADP and thus suppressing the expression of inflammatory markers [56, 57]. Evidence from in vitro studies has also shown that folic acid may reduce inflammation by inhibiting the phosphoinositide 3-kinases (PI3K)/hypoxia-inducible factor 1-alpha (HIF-1α) pathway [58]. Even though the reduction in homocysteine following folate supplementation is normally accompanied by a reduction in CRP and subsequent deactivation of NF-κβ and low IL-6 and TNF-α, the contradictory findings observed in our study may be due to few trials and sample size across the trials analyzed in this study.

### Strength and limitation

The present analyses exclusively examined evidence from randomized trials, considered to provide high clinical evidence. Notably, there was a low risk of bias observed across various domains in the risk of bias assessment, indicating that the quality of the studies was satisfactory. The GRADE tool was also employed to evaluate the overall quality of the analyzed evidence, and it was categorized as either moderate or very low in one outcome due to the small sample size.

Furthermore, a comprehensive subgroup analysis was performed, considering various confounding factors. The *I*^2^ statistics revealed moderate heterogeneity. For transparency, the study was registered with PROSPERO, and the experienced researchers adhered to PRISMA guidelines, boosting confidence in the reliability of the current findings. However, it is crucial to acknowledge certain limitations in our study, such as few relevant trials, indicating a minimal sample size of only 426 patients living with T2DM. Moreover, existing trials have employed varying quantitative methodologies, introducing potential differences in sensitivity and specificity, especially with the use of ELIZA AND HPLC for the determination of homocysteine.

## Conclusion and future recommendations

The findings from nine trials involving a sample of 426 participants in this study indicate that folic acid supplementation in T2DM may reduce homocysteine levels, a potential biomarker for CVDs. However, due to the limited number of trials analyzed, null effects were observed concerning some of the inflammatory markers. It is crucial to interpret the conclusions of our study with caution, emphasizing the need for further trials with adequate sample sizes.

Considering the limitations acknowledged in this study, we propose recommendations for future investigations into folic acid in T2DM, particularly focusing on inflammation. We suggest that forthcoming RCTs use sufficient sample sizes and adhere to the reporting guidelines outlined in the consolidated standards of reporting trials (CONSORT). Additionally, these trials should adhere to standardized methodologies, implementing an accurate randomization process, blinding of personnel and participants. Furthermore, we emphasize the necessity for high-quality meta-analyses to comprehensively elucidate the benefits of folic acid supplementation in managing T2DM.

### Supplementary information

Appendix 2

## Data Availability

The data used in this review are available from the corresponding author upon reasonable request.
