# Supplementary material for: Folic acid supplementation on inflammation and homocysteine in type 2 diabetes mellitus: systematic review and meta-analysis of randomized controlled trials
Source: Nutr Diabetes. 2024 Apr 22;14:22. doi: 10.1038/s41387-024-00282-6 (PMC11035602; doi:10.1038/s41387-024-00282-6)
Supplement: Supplementary file 1 — Appendix 2 [file 41387_2024_282_MOESM1_ESM.docx]

**Folic acid Supplementation on Inflammation and** **Homocysteine in Type 2 Diabetes Mellitus: Systematic Review** **and Meta-analysis of Randomized Controlled Trials**

**Running title: Folic acid in type 2 diabetes mellitus**

Kabelo Mokgalaboni^1*^, Given. R Mashaba^1^, Wendy N. Phoswa^1^, Sogolo. L Lebelo^1^

^1^Department of Life and Consumer Science, College of Agriculture and Environmental Sciences, University of South Africa, Florida Campus, Roodepoort, 1710, South Africa.

*Corresponding author

Mr Mokgalaboni Kabelo

Emails: [mokgak@unisa.ac.za](mailto:mokgak@unisa.ac.za);

Department of Life and Consumer Sciences

University of South Africa

Calabash Building Office no 02-047

Florida, 1710

**Table legends**

Table 1S: Search strategies used on database.

Table 2S: Sensitivity analysis results on homocysteine

Table 3S: Sensitivity analysis results on CRP

Table 4S: Sensitivity analysis results on TNF-α

Table 5S: Sensitivity analysis results on IL-6

Table 6S: GRADE results on the effect of folate on homocysteine and inflammation

**Figure legends**

Figure 1S: Subgroup analysis on homocysteine based on sample size and dosage. A. Subgroup analysis based on sample size; B. subgroup based on the dosage of folic acid

Figure 2S: Subgroup analysis on homocysteine based on gender and duration of intervention.

Figure 3S: Subgroup analysis on CRP in type 2 diabetes mellitus

**Table 1S:** Search strategies used on database.

| **PubMed** |  |
| --- | --- |
| **MESH TERMS** | **HITS** |
| Folate | 42,187 |
| Folic acid | 42,137 |
| Folacin | 42,187 |
| Vitamin B9 | 42,187 |
| Homocysteine | 17,037 |
| Inflammation | 421,584 |
| Type 2 diabetes mellitus | 174,205 |
| ((((((Folate[MeSH Terms]) OR (Folic acid[MeSH Terms])) OR (folacin[MeSH Terms])) OR (vitamin b9[MeSH Terms])) AND (homocysteine[MeSH Terms])) AND (Inflammation[MeSH Terms])) AND (type 2 diabetes mellitus[MeSH Terms]) | 2 |
| **Scopus** |  |
| (TITLE-ABS-KEY ( folate ) OR TITLE-ABS-KEY ( folic AND acid ) OR TITLE-ABS-KEY ( vitamin AND b9 ) OR TITLE-ABS-KEY ( folacin ) AND TITLE-ABS-KEY ( homocysteine ) AND TITLE-ABS-KEY ( inflammation ) AND TITLE-ABS-KEY ( type 2 diabetes AND mellitus ) | 20 |
| **Cochrane Library** |  |
| MESH descriptor [Folic acid] AND MESH descriptor [type 2 diabetes] | 26 |

**
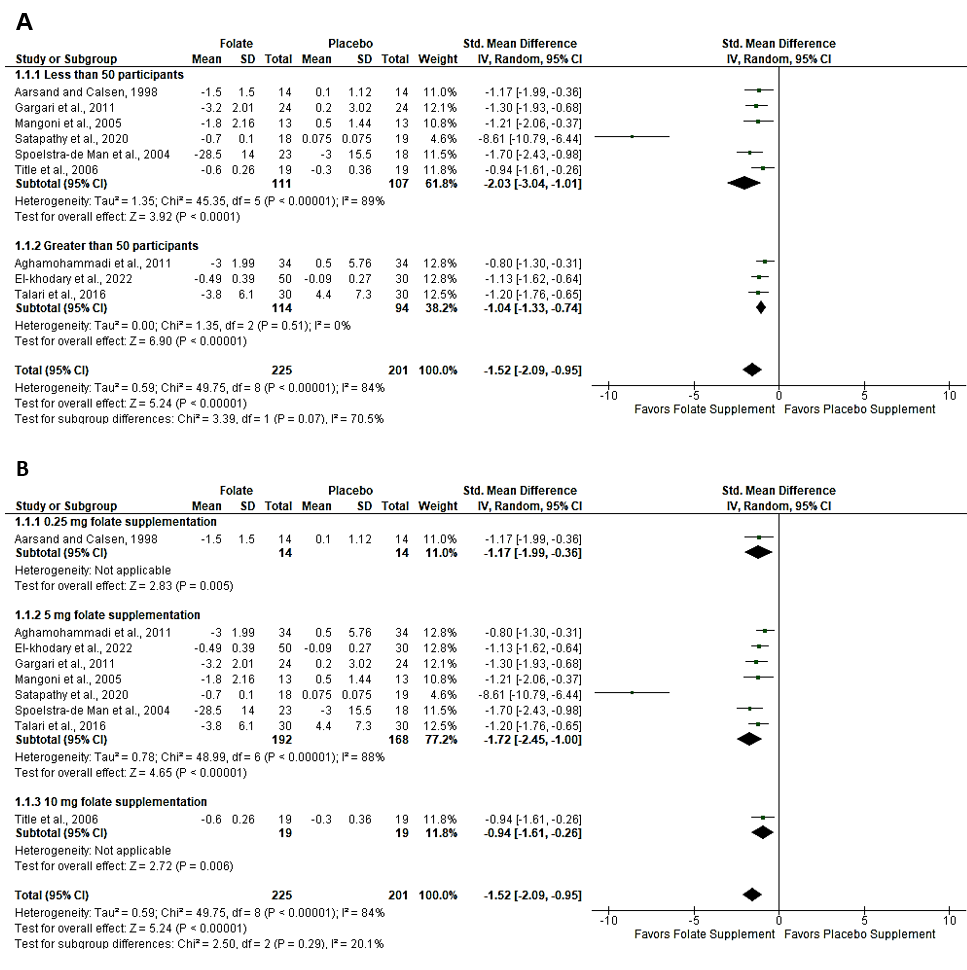
**

**Figure 1S:** Subgroup analysis on homocysteine based on sample size and dosage. A. Subgroup analysis based on sample size; B. subgroup based on the dosage of folate


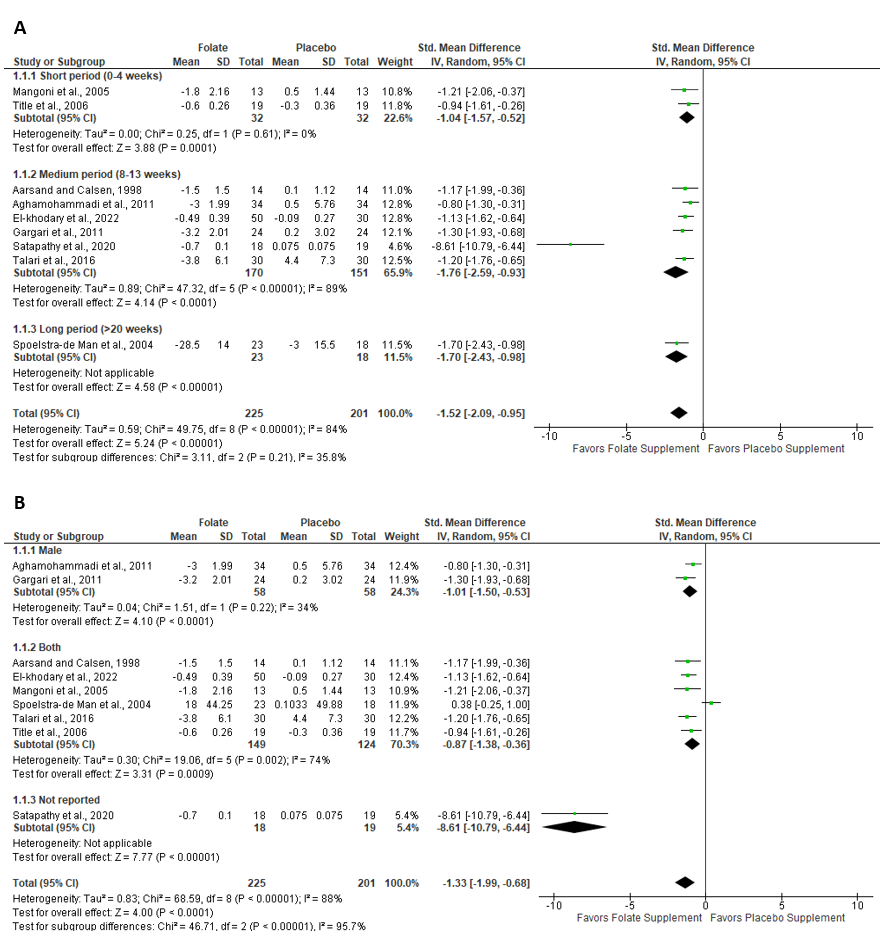


**Figure 2S:** Subgroup analysis on homocysteine based on gender and duration of intervention.

**
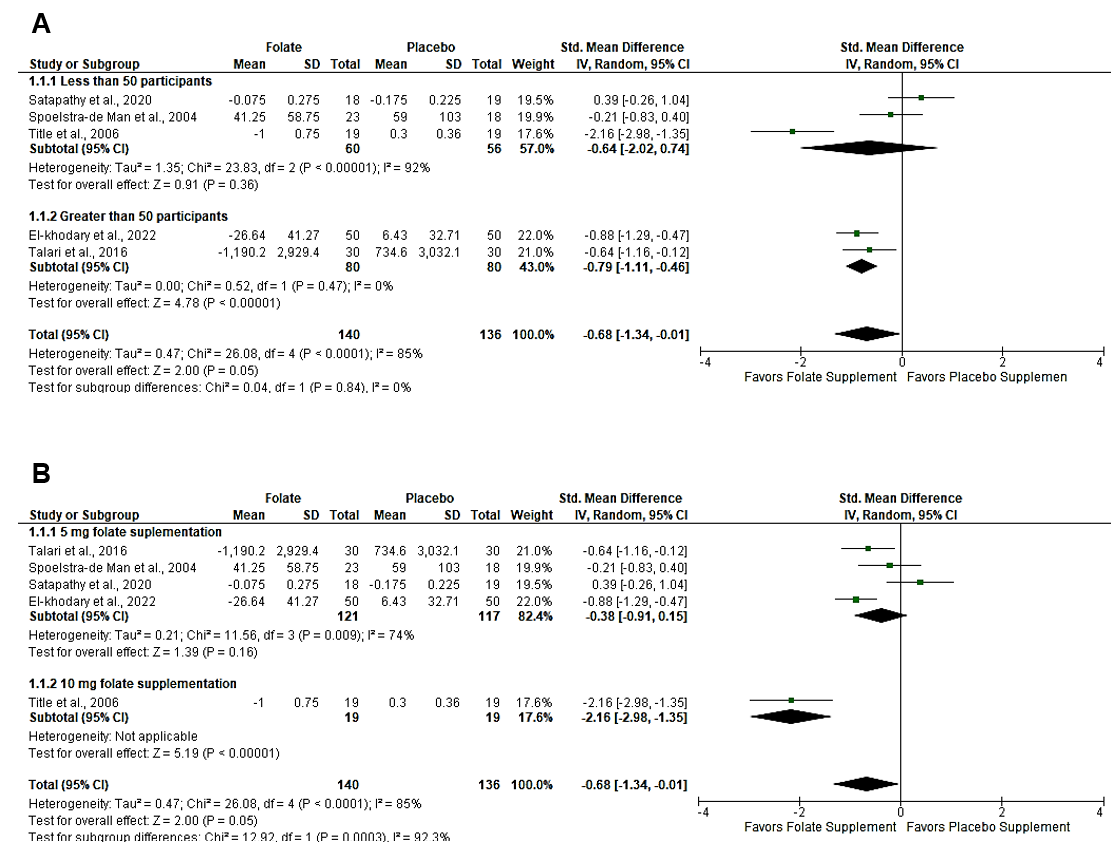
**

**Figure 3S:** Subgroup analysis on CRP in type 2 diabetes mellitus

**Table 2S**: Sensitivity analysis for results on homocysteine

| **Deleted Study** | **Effect Size** | **Lower CI** | **Upper CI** | **P Value** | **I Square** |
| --- | --- | --- | --- | --- | --- |
| Aarsand and Calsen, 1998 | -1.9790 | -3.5810 | -0.3771 | 0.0155 | 97.9422 |
| Aghamohammadi et al., 2011 | -2.0262 | -3.6117 | -0.4406 | 0.0123 | 97.5890 |
| El-khodary et al., 2022 | -1.9866 | -3.5909 | -0.3823 | 0.0152 | 97.6297 |
| Gargari et al., 2011 | -1.9646 | -3.5739 | -0.3552 | 0.0167 | 97.8366 |
| Mangoni et al., 2005 | -1.9739 | -3.5769 | -0.3710 | 0.0158 | 97.9605 |
| Satapathy et al., 2020 | -1.1403 | -1.3566 | -0.9239 | 0.0000 | 0.0000 |
| Spoelstra-de Man et al., 2004 | -1.9142 | -3.5296 | -0.2987 | 0.0202 | 97.9305 |
| Talari et al., 2016 | -1.9774 | -3.5840 | -0.3707 | 0.0159 | 97.7421 |
| Title et al., 2006 | -2.0088 | -3.6010 | -0.4166 | 0.0134 | 97.8319 |

**Table 3S:** Sensitivity analysis results on CRP

| **Deleted Study** | **Effect Size** | **Lower CI** | **Upper CI** | **P Value** | **I Square** |
| --- | --- | --- | --- | --- | --- |
| Title et al., 2006 | -0.3886 | -0.8736 | 0.0963 | 0.0998 | 65.9574 |
| Talari et al., 2016 | -0.6963 | -1.6270 | 0.2345 | 0.1245 | 88.6113 |
| Spoelstra-de Man et al., 2004 | -0.7986 | -1.6787 | 0.0814 | 0.0623 | 88.4689 |
| Satapathy et al., 2020 | -0.9251 | -1.5893 | -0.2610 | 0.0042 | 80.1355 |
| El-khodary et al., 2022 | -0.6302 | -1.5594 | 0.2991 | 0.1637 | 87.1244 |

**Table 4S:** Sensitivity analysis results on TNF-α

| **Deleted Study** | **Effect Size** | **Lower CI** | **Upper CI** | **P Value** | **I Square** |
| --- | --- | --- | --- | --- | --- |
| Satapathy et al., 2020 | 0.1952 | -0.2709 | 0.6612 | 0.3897 | 0.0000 |
| Spoelstra-de Man et al., 2004 | -1.5052 | -3.7515 | 0.7411 | 0.1688 | 92.8380 |
| Title et al., 2006 | -1.3176 | -3.8417 | 1.2066 | 0.2837 | 94.4002 |

**Table 5S:** Sensitivity analysis results on IL-6

| **Deleted Study** | **Effect Size** | **Lower CI** | **Upper CI** | **P Value** | **I Square** |
| --- | --- | --- | --- | --- | --- |
| Satapathy et al., 2020 | 0.4940 | -0.1320 | 1.1200 | 0.1219 | 0.0000 |
| Spoelstra-de Man et al., 2004 | -0.5753 | -1.2332 | 0.0825 | 0.0865 | 0.0000 |

**Table 6S:** GRADE results on the effect of folate on homocysteine and inflammation

| **Outcomes** | **№ of participants (studies) Follow-up** | **Certainty of the evidence (GRADE)** | **Relative effect (95% CI)** | **Anticipated absolute effects** | |
| --- | --- | --- | --- | --- | --- |
|  |  |  |  | **Risk with placebo** | **Risk difference with folate and placebo** |
| Homocysteine assessed with: µmol/L | 426 (9 RCTs) | ⨁⨁⨁◯ Moderate^a,b,c^ | - | - | SMD **1.52 SD lower** (2.09 lower to 0.95 lower) |
| C-Reactive Protein (CRP) | 276 (5 RCTs) | ⨁⨁⨁◯ Moderate^d,e,f,g^ | - | - | SMD **0.68 SD lower** (1.34 lower to 0.01 lower) |
| Tumor Necrosis Factor-Alpha (TNF-α) | 116 (3 RCTs) | ⨁⨁⨁◯ Moderate^b,d,e,h^ | - | - | SMD **0.86 SD lower** (2.65 lower to 0.93 higher) |
| Interleukin-6 (IL-6) | 78 (2 RCTs) | ⨁◯◯◯ Very low^b,d,i,j^ | - | - | SMD **0.04 SD lower** (1.08 lower to 1.01 higher) |
| ***The risk in the intervention group** (and its 95% confidence interval) is based on the assumed risk in the comparison group and the **relative effect** of the intervention (and its 95% CI).  **CI:** confidence interval; **SMD:** standardised mean difference | | | | | |
| **GRADE Working Group grades of evidence** **High certainty:** we are very confident that the true effect lies close to that of the estimate of the effect. **Moderate certainty:** we are moderately confident in the effect estimate: the true effect is likely to be close to the estimate of the effect, but there is a possibility that it is substantially different. **Low certainty:** our confidence in the effect estimate is limited: the true effect may be substantially different from the estimate of the effect. **Very low certainty:** we have very little confidence in the effect estimate: the true effect is likely to be substantially different from the estimate of effect. | | | | | |

**Explanations**

a. Two trials analyzed were judged as some concern of bias in two domains

b. evidence of publication bias was observed graphically through funnel plot and statistically through Eggers regression test (p value less than 0.05)

c. The effect size was large, with SMD of 1.52 which is large effect according to Cohens d

d. All trials analyzed showed no risk of bias and thus were judged as low risk of bias according to Cochrane risk of bias

e. Small sample size

f. The funnel plot and Eggers regression test showed no evidence of publication bias

g. The effect size is ranging from small to medium, with SMD of 0.68 which is medium effect according to Cohens d

h. Large effect size, with SMD of 0.86 which is large according to Cohens d

i. Very small sample size (78) participants

j. Small effect size, with SMD of 0.04 according to Cohens d
